# Digital Gene Expression Analysis of *Microsporum canis* Exposed to Berberine Chloride

**DOI:** 10.1371/journal.pone.0124265

**Published:** 2015-04-14

**Authors:** Chen-Wen Xiao, Quan-An Ji, Qiang Wei, Yan Liu, Li-Jun Pan, Guo-Lian Bao

**Affiliations:** Institute of Animal Husbandry and Veterinary Science, Zhejiang Academy of Agricultural Sciences, Hangzhou, Zhejiang Province, China; University Paris South, FRANCE

## Abstract

Berberine, a natural isoquinoline alkaloid of many medicinal herbs, has an active function against a variety of microbial infections including *Microsporum canis (M*. *canis)*. However, the underlying mechanisms are poorly understood. To study the effect of berberine chloride on *M*. *canis* infection, a Digital Gene Expression (DGE) tag profiling was constructed and a transcriptome analysis of the *M*. *canis* cellular responses upon berberine treatment was performed. Illimina/Hisseq sequencing technique was used to generate the data of gene expression profile, and the following enrichment analysis of Gene Ontology (GO) and Pathway function were conducted based on the data of transcriptome. The results of DGE showed that there were 8476945, 14256722, 7708575, 5669955, 6565513 and 9303468 tags respectively, which was obtained from *M*. *canis* incubated with berberine or control DMSO. 8,783 genes were totally mapped, and 1,890 genes have shown significant changes between the two groups. 1,030 genes were up-regulated and 860 genes were down-regulated (P<0.05) in berberine treated group compared to the control group. Besides, twenty-three GO terms were identified by Gene Ontology functional enrichment analysis, such as calcium-transporting ATPase activity, 2-oxoglutarate metabolic process, valine catabolic process, peroxisome and unfolded protein binding. Pathway significant enrichment analysis indicated 6 signaling pathways that are significant, including steroid biosynthesis, steroid hormone biosynthesis, Parkinson’s disease, 2,4-Dichlorobenzoate degradation, and tropane, piperidine and Isoquinoline alkaloid biosynthesis. Among these, eleven selected genes were further verified by qRT-PCR. Our findings provide a comprehensive view on the gene expression profile of *M*. *canis* upon berberine treatment, and shed light on its complicated effects on *M*. *canis*.

## Introduction

Dermatophytes are pathogenic fungi that have the ability to invade the keratinized structure and infect skin, hair and nails of human and animals [1]. The genera *Microsporum canis* (*M*. *canis*) was found in both man and animal and the species *M*. *canis* is zoonotic in nature. Previous studies revealed that *M*. *canis* is the major cause of dermatophytosis in pets and rabbits. In total, 21 isolates of *M*. *canis* were collected from rabbits with or without skin lesions [1]. Although there are a variety of drugs, including azoles, propylene amine, ring ketone amine, and amorolfine, the overall effect is not stable [2]. Therefore, discovery of novel effective medicine against rabbit dermatosis is urgent. The interest in natural medicine has remarkably increased in recent years due to the side effects of conventional drugs, as well as the emergence of antimicrobial resistance to available drugs. Plants and their derivatives are therefore an important alternative in the development of novel drugs. The bark from the Phellodendron tree has been used as traditional Chinese medicine for thousands of years. The species, *Phellodendron amurense*, is widely used to treat gastroenteritis, abdominal pain or diarrhea, and various inflammatory diseases including arthritis and dermatophytosis [3]. Recently the ethanol extract from *Phellodendron amurense* has been found to inhibit the growth of certain kinds of fungi such as *Trichophyton mentagrophytes* [4] and *Staphylococcus* [5], and in our previously experiment, the MIC value of berberine against *M*. *Canis* was 2 mg/mL. Berberine, a major component of *M*. *Canis*, is a natural isoquinoline alkaloid found in medicinal herbs. Previous studies have implied a number of biological activities of berberine, including anti-secretory, anti-inflammatory, anti-bacterial, anti-malarial, anti-mycobacterial [6], anti-tumor and anti-cholesterol activities. Therefore, berberine has been widely used in the treatment of bacterial diarrhea and intestinal parasite infections in many countries.

The digital gene expression (DGE) system is a tag-based transcriptome sequencing approach in the production of technical innovation of RNA deep sequencing technologies, which is mainly used to study the comparative gene expression. When DGE is applied, clean tags strained through raw tags with certain end nuclease should be mapped to the reference database. The degrees of expression of the actual total genes in the sample are defined by the quantity of each mRNA yield from each gene. Therefore, in this study, by using DGE, we performed a large-scale analysis of gene expression changes when *M*. *canis* was exposed to berberine, to better understand how berberine inhibits the growth of *M*. *canis*.

## Materials and Methods

### Fungi growth and berberine hydrochloride treatment

The *M*. *canis* strain (CMCC(F)M3h) used in this study was provided by the Institute of Internal Medicine, Chinese Academy of Medical Sciences (Nanjing, China) National Center. The *M*. *canis* was cultured in Tryptic Soy Broth (TSB) at 28°C for 96 h. The densities of the suspension were adjusted to primary inoculum of 1.0 × 10^7^ CFU/mL in the normal saline solution with total volume of 100 mL in conical flasks. Berberine hydrochloride (BC) (HPLC>98%) was purchased from Yuan Ye Biological technology co., Ltd, Shanghai, China. The BC treated group (three samples) was subject to medicinal stress dose which means 1/2 MIC (minimum inhibitory concentration) dose (1 mg/mL berberine in DMSO), while the control group was fed with DMSO (Purity>99%) which was purchased from Sangon Biotech co., Ltd Shanghai, China only. Each group has three repeats. After 6 h incubation (28°C,150 rpm), the seeds were filtered with gauze and frozen after treatment for RNA isolation and further analysis.

### RNA isolation and library preparation for DGE

Total RNA was extracted using Trizol reagent (Invitrogen, CA, USA) following the manufacturer’s procedure. Total RNA quantity and purity were analyzed by Bioanalyzer 2100 and RNA 6000 Nano LabChip Kit (Agilent, CA, USA) with RIN number >8.0. Approximately 10 μg of total RNA from each sample was subjected to isolate Poly (A) mRNA by using poly-T oligo attached magnetic beads (Invitrogen). Following purification, the mRNA is fragmented into small pieces using divalent cations under elevated temperature. Then the cleaved RNA fragments were reverse-transcribed to create the final cDNA library in accordance with the protocol for the mRNA-Seq sample preparation kit (Illumina, San Diego, USA), the average insert size for the single-end libraries was 200bp-350bp. Then we performed the single-end sequencing (36 bp) on an Illumina Hiseq2500 at the LC-BIO (Hangzhou, China) following the vendor’s recommended protocol. Data has been deposited to the server of National Institute of Health (NIH) (http://trace.ncbi.nlm.nih.gov/Traces/sra_sub/sub.cgi?subid=298920&from=list&action=show:submission), and included the accession number (SRP044202).

### DGE tag profiling

10μg purified mRNA of each sample was used for synthesizing the first- and second-strand cDNA. A library containing all the possible fragments of CATG+17 bases in length among the reference gene sequences was generated. All clean tags were mapped to the reference sequences allowing for only 1 bp mismatch. Clean tags that mapped to reference sequences from multiple genes were filtered. The remaining clean tags were designed as unambiguous clean tags, the number of which was calculated for each gene and normalized to the number of transcripts per million clean tags (TPM).

### Analysis of differential gene expression

The detection of differential gene expressions across samples was performed using a rigorous algorithm method [7] and the differences of gene expression profile between berberine-treatment group and control group were calculated and compared by DESeq software which is good for differential expression analysis in biological samples. False discovery rate (FDR) was used to determine the *P value* threshold in multiple tests and analyses. For our experiment, we had three repeats for each sample group, and we only considered the significant different gene expression with a P value equal or less than 0.05 and the value of |log 2 ratio | ≥1, which means that the fold change of gene expression between berberine-treatment group and control group is equal to or greater than 2.

### Gene ontology and Kyoto Encyclopedia of Genes and Genomes

The gene ontology (GO) classification system was used to determine the possible functions of all differentially expressed genes. *P-value* was calculated for each changed gene. A corrected *P-value* ≤0.05 was selected as a threshold for significant enrichment of the gene sets. Pathway enrichment analysis can further identify significantly enriched metabolic pathways or signaling transduction pathways using the Kyoto Encyclopedia of Genes and Genomes (KEGG) database. Pathways with a *P-value* ≤0.05 are considered as significantly enriched pathways in DGE. GO enrichment analysis of functional significance applies a hypergeometric test to map all DEGs to the GO database, identifying significantly enriched GO terms compared to the genomic background. Pathway analysis was mainly based on the KEGG database. Two-sided Fisher’s exact tests with multiple testing and the X^2^ test were used to classify the pathway categories. The FDR was used to correct *P* values, and only pathway categories with *P* ≤0.05 were chosen.

### Real-time PCR analysis

One μg of total RNA from each sample was used to synthesize the first strand cDNA using the kit from Promega (Madison, WI, US) according to the protocol of the manufacturer. Quantitative RT-PCR was carried out in an ABI StepOnePlus (Applied Biosystems) using SYBR Green Supermix (TaKaRa) according to manufacturer instruction. The thermal cycle conditions were 94°C for 10 min for denaturation, followed by 40 cycles of 94°C for 15 s, and 60°C for 31 s for annealing, and then an extension. The primers of examined genes are listed in Table 1. Each gene was analyzed in triplicate, and the average threshold cycle (CT) was calculated by 2−ΔΔCt method [8], the comparative Ct method we used in the manuscript is known as the 2-ΔΔCt method, where ΔΔCt = ΔCt, sample -ΔCt, reference. Here ΔCt, sample is the Ct value for any sample (berberine-treated group) normalized to the endogenous housekeeping gene and ΔCt, reference is the Ct value for the calibrator (control group) also normalized to the endogenous housekeeping gene. The expression of 18S was as an endogenous housekeeping control. The value stood for the fold difference relative to the calibrator. All data were given in terms of relative mRNA expression as mean ± SD.

**Table 1:** The primer in real-time PCR.

Gene Name	Primer	Sequences(5'-3’)
cytochrome P450	F	GCAGGAACCAATATTAACGCC
	R	TTGTATTGCAGGGGAGCCAG
Na+/H+ antiporter	F	ACGGTCTCGTGCCAAAACTA
	R	GTACCCCAGTCCGTCATTAGC
lipase 2	F	AGCAGCTATCGCCTCCTCTA
	R	GAAGCAAGGGGGCTGAAAG
zinc transporter zupT	F	CTACCTTACTCGGGCTGGTTACT
	R	TGGTGTGCTGCTATGCTGAT
glycerol-3-phosphate phosphatase	F	AAAGGACCTTGGAACTGATGC
	R	GACACCCGAGGGAGCGT
multicopper oxidase	F	CTGGACTGTCTATCCCGCAC
	R	TGAGCATTTGGAGCCTGTGT
maleylacetoacetate isomerase	F	AACCCTTCCGAACACTCGTC
	R	TCGTCACAGGCTGAATGTCC
succinyl-CoA:3-ketoacid-coenzyme A transferase	F	CGGTGGAGGTACAGCTTCAG
	R	GAGAGTGACGGTCTCCTTGC
NADPH-dependent D-xylose reductase	F	TCGAGTTCTGATCGCATGGC
	R	TCGCGGACTGAGAGAGTTCA
Ribonucleoprotein	F	GTCCAGGAACTCTTCTCCAAGCACG
	R	GCCACCAAGGTCAGCACCGTATT
symbiotic chitinase	F	CTACAGTGGGATACGACCGAGC
	R	TTCCCACCGCGACTGCA
18s rRNA	F	TGGTGCATGGCCGTTCTTA
	R	GGTCTCGTTCGTTATCGCAATT

### Statistic analysis

All data were analyzed using a *Duncan’s test* with a minimum n = 3. P-values equal to or less than 0.05 were considered statistically significant.

## Results

### Analysis of DGE libraries

We obtained 8476945 clean reads from control group 1 (C1), 14256722 clean reads from control group 2 (C2), 7708575 clean reads from control group 3 (C3), 5669955 clean reads from BC-treated group 1 (X1), 9303468 clean reads from BC-treated group 2 (X2) and 6565513 clean reads from BC-treated group 3 (X3). Among them, 742523 (C1), 2392248 (C2), 483790 (C3), 152935 (X1), 424366 (X2), and 172032 (X3) were greater than 100 copies respectively (Table 2). We also generated 3128151 (C1), 3464239 (C2), 3007891 (C3), 2758573 (X1), 3441368 (X2), and 3159984 (X3) unique reads respectively. Among the unique reads, 3868 (C1), 10684 (C2), 2744 (C3), 940 (X1), 1852 (X2), and 1082 (X3) reads were greater than or equal to 100 copies respectively. The results showed that the number of detected genes was almost saturated when the total tag number reached 4 million or higher. Our sequencing depths reached approximately 5 million in each DGE library, which met the requirement for the experiment.

**Table 2:** Summary of DGE sequences analysis.

	C1	C2	C3	X1	X2	X3
Raw Reads	8513165	14319720	7750073	5691651	9339451	6591810
Clean Reads	8476945	14256722	7708575	5669955	9303468	6565513
Total mapped	6272409	10510592	5928905	4339331	6708360	4987496
mapped gene	8532	8553	8557	8561	8621	8595
Match(uniqe Sense)< = 1 mismatch						
1 unique seq-> 1 gene	6212660	10412914	5867963	4280753	4927102	6607106
1 unique seq-> n gene	15260	26590	16007	11525	10853	18131
Match(uniqe Antiense)< = 1 mismatch						
1 unique seq-> 1 gene	44407	70915	44838	46949	49433	82980
1 unique seq-> n gene	13	50	27	38	35	51
Match(both Sense and Antiense)< = 1 mismatch						
1 unique seq-> n genes	69	123	70	66	73	92

### GO analysis and KEGG pathways

**Fig 1:**
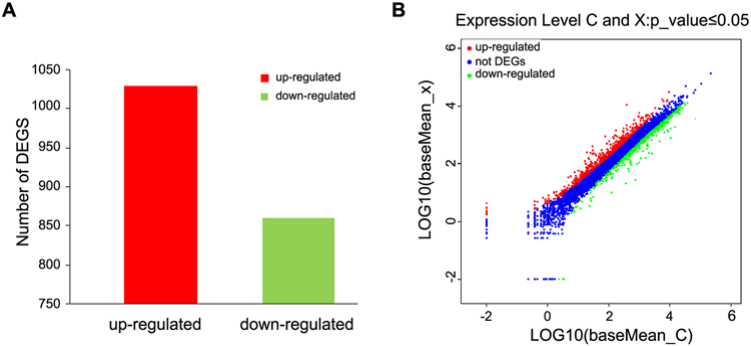
Differentially-expressed gene (DEGs) in different groups (*P ≤0*.*05*). A. Totally 1890 differentially expressed genes were detected, of which 1030 were up-regulated and 860 were down-regulated (*P ≤0*.*05*). B. Differentially expressed genes. The red part represents the up-regulated genes in berberine hydrochloride-treated group compared to control group. The green part indicates the down-regulated genes in berberine hydrochloride-treated group. The blue part shows the genes without expression difference between these two samples.

**Fig 2:**
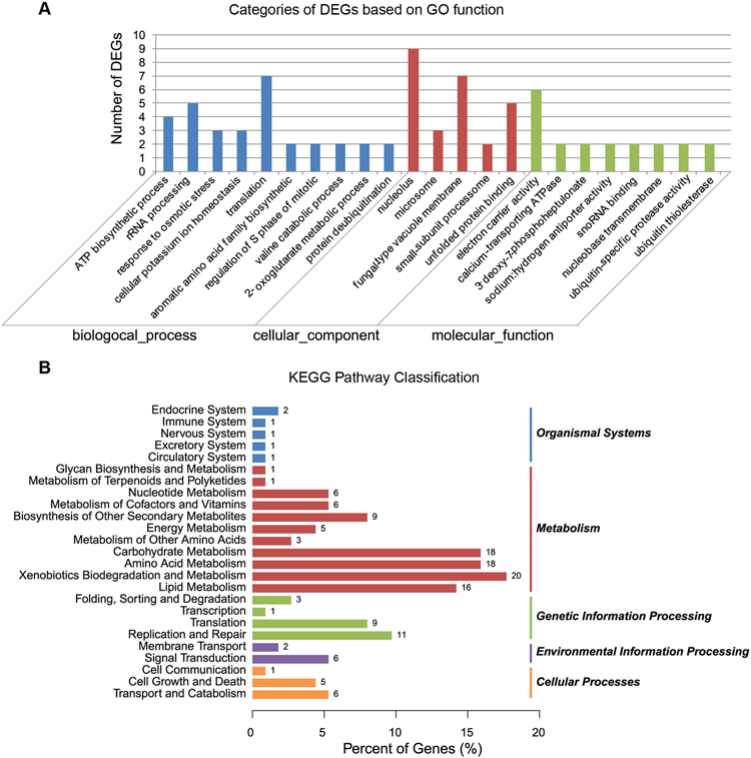
Histogram diagram of Gene Ontology classification. A. Categories of DEGs based on GO function. The results are summarized in three major categories: biological process, cellular component and molecular function. The y-axis on the left indicates the number of genes in a category. B. KEGG pathway classification of identified genes.

A total of 1890 differentially expressed genes were detected, of which, 1030 were up-regulated and 860 were down-regulated in berberine-treated samples (Fig 1A and 1B). To understand their functions, all of the 1890 differentially expressed genes were mapped to terms in GO database and compared with the whole genome background. GO has three ontologies, namely, molecular function, cellular component, and biological process. 78 differentially expressed genes including 35 up-regulated genes and 43 down-regulated genes have a GO ID and can be categorized into a total of 23 functional groups using the WEGO software [9] (Fig 2A and 2B) (S1 Dataset).

Out of the three main categories of the GO classification, ‘‘biological process” is dominant. Interestingly, some genes, related to the rRNA processing, translation, and nucleolus, were all down-regulated in berberine-treated samples (Fig 2A). KEGG (http://www.genome.jp/kegg) ontology assignments were used to classify the functional annotations of the identified genes to further understand their biological functions (Fig 2B). Among the differentially expressed genes, 128 were mapped to 76 pathways in the KEGG database in *Microsporum canis* DGE class, and six terms were significantly enriched (*P*≤0.05) (S2 Dataset).

### Analysis of tag mapping

In the control (C1, C2, C3) and berberine-treated samples (X1, X2, X3) DGE library, 73.99%, 73.72%, 76.91%, and 76.53%, 72.11%, 75.97% of the clean tags corresponding to 64.98%, 61.75%, 71.89%, and 71.69%, 67.18%, 70.54% of distinct tags were mapped to a gene in the reference database respectively.

### Identification and verification of differentially expressed genes

**Fig 3:**
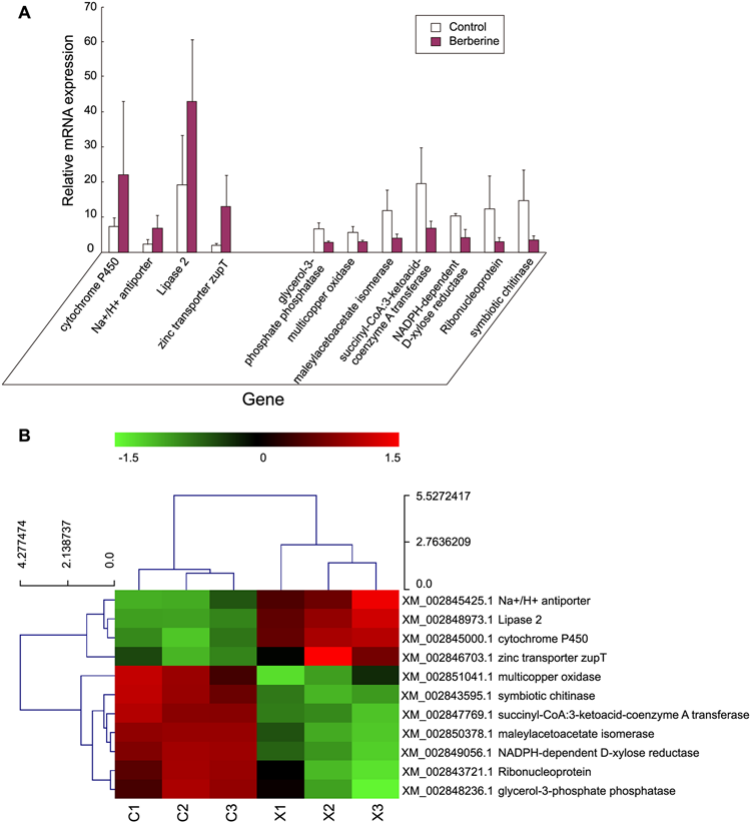
Differential expression levels of candidate genes in berberine hydrochloride-treated and control of *Microsporum canis*. A. Six biological replicates were used, and 18s rRNA was used as an internal control. The y-axis indicates the relative expression level of candidate gene mRNA transcripts (Fold change = log2 Ratio). The x-axis indicates the candidate genes (from left to right) with the names of cytochrome P450 (CYP450), Na^+^/H^+^ anti-porter (Na anti-porter), lipase 2 (L2), zinc transporter zupT (zt zupT), glycerol-3-phosphate phosphatase (G3P), multicopper oxidase (MO), maleylacetoacetate isomerase (MI), succinyl-CoA:3-ketoacid-coenzyme A transferase (SCT), NADPH-dependent D-xylose reductase (NADPHR), ribonucleoprotein (RNP), and symbiotic chitinase (sc). The y-axis is the fold-change between berberine hydrochloride-treated and control samples (Fold change = log2 Ratio for real-time PCR and DGE). Means with the star on the column differ significantly (*P<0*.*05*). B. Heat map of selected identified genes (*P ≤0*.*05*) Hierarchical clustering of differentially expressed genes in Berberine treatment samples (X1,X2,X3) compared with the Control sample (C1,C2,C3). Red indicates up-regulation, and green indicates down-regulation.

The differentially expressed tags between the two samples were identified by *P* ≤0.05. A total of 1860 significantly changed genes were found with 1030 up-regulated and 860 down-regulated genes in DGE libraries (Fig 1). A total of 11 differentially expressed genes including 4 up-regulated and 7 down-regulated genes from DGE libraries were selected for real-time PCR analysis to validate the DGE data. The results showed that 11 genes were demonstrated to have a consistent change for both DGE and real-time PCR while cytochrome P450, lipase 2, zinc transporter zupT and symbiotic chitinase genes had no significant difference in real-time PCR (*P* >0.05) (Fig 3A and 3B).

## Discussion

Efficiency of medicinal plants against fungi has been reported [2, 4], but studies on their underlying mechanisms are very few. Berberine chloride, a member of alkaloids and the major bioactive component of *Cortex phellodendri*, has been reported to display various biological and pharmacological activities [10]. Park etc. has reported that berberine could inhibit *Candida krusei* with MIC <1 μg/ml [11]. To determine the anti-fungal mechanism of *Phellodendron amurense* against *M*. *canis*, we performed *in vitro* experiments with berberine chloride. To comprehensively evaluate gene profile, we performed a large-scale analysis of gene expressions in *M*. *canis* upon berberine chloride treatment using DGE approach. KEGG analysis suggested that several signaling pathways are affected in *M*. *canis* upon berberine chloride treatment, including metabolism, genetic information processing, environmental information processing, cellular processes, and organismal system. The results of qRT-PCR are consistent with DGE data, confirming that changes of gene expression upon berberine chloride treatment.

Most genes (32 genes) identified were connected to cellular metabolism, biosynthesis, and stress responses. Among these genes, glycerol-3-phosphate phosphatase (XM_002848236.1) (GPPs) and Na^+^/H^+^ anti-porter (XM_002845425.1) were down-regulated and up-regulated respectively upon berberine treatment. The hydrolysis of glycerol-3-phosphate to glycerol by GPPs is important for hyperosmotic stress responses, as described in the eukaryotes *Saccharomyces cerevisiae* and *Candida albicans* [12, 13]. Our data suggested that increased expression of GPPs in *M*. *canis* by berberine treatment might be one of the anti-fungal mechanisms. Berberine may modulate the expression of GPPs to weaken the responses to hyperosmotic stresses, which leads to dramatic changes in circumstances around *M*. *canis* and the following apoptosis.

The phenomenon of up-regulation of Na^+^/H^+^ anti-porter was consistent with the results of Wang etc. [14] Na^+^/H^+^ anti-porters play a key role in maintenance of the cytoplasmic K^+^/Na^+^ ratio, through pumping Na^+^ either into or out of organelles, mainly vacuole, to exchange for H^+^. The over expression of Na^+^/H^+^ anti-porter genes could increase tolerance under saline conditions in buckwheat [15] and *Petunia hybrida* [16]. Up-regulation of Na^+^/H^+^ anti-porter from our experiments suggested that *M*. *canis* might modulate the expression of Na^+^/H^+^ anti-porter genes to tolerate the berberine exposure.

We also identified that heavy metal tolerance protein (XM_002850584.1), a member of ATP-binding cassette (ABC) transporter pathway, is down-regulated upon berberine treatment. ABC transporters constitute one of the largest protein families in nearly all the living organisms, and are involved in some detoxification processes, which help cell recovery from various stress conditions. Our results suggested that berberine treatment limited ABC transporter-related gene expressions to increase berberine accumulation in *M*. *canis*, leading to the death of *M*. *canis*.

**Fig 4:**
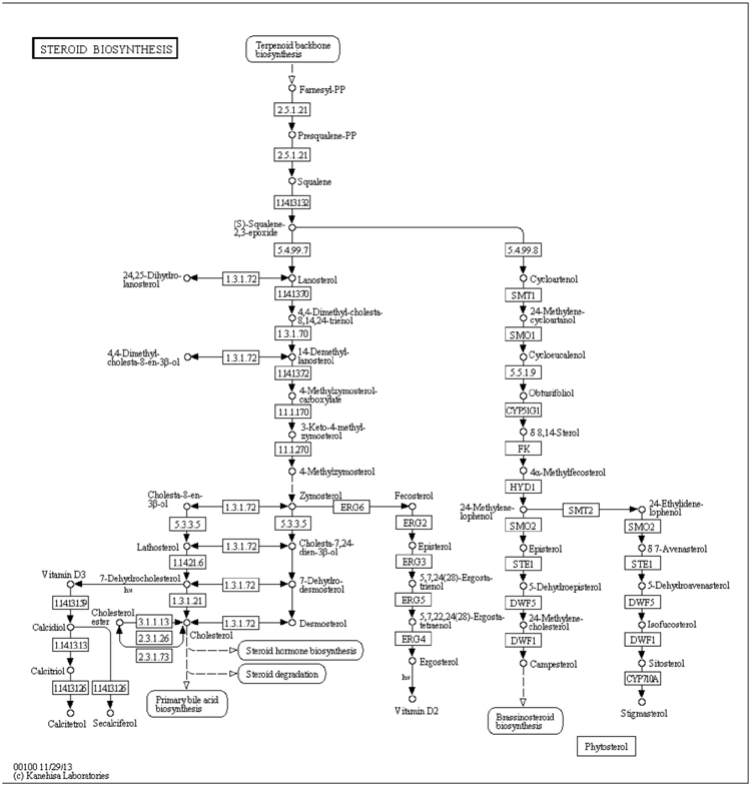
The KEGG pathway of steroid biosynthesis. CYPs function in the metabolism of aliphatic, alicyclic, and aromatic molecules to regulate hydroxylation, epoxidation, dealkylation, sulfoxydation, deamination, desulphuration, dehalogenation, and noxide reduction. In present research, Cytochrome P450 was up-regulated upon berberine chloride treatment, which suggested that it might be involved in tolerance of toxic chemicals.

Another important identified KEGG pathway is the steroid biosynthesis pathway. Among the genes involved in steroid biosynthesis pathway (Fig 4), cytochrome P450 enzymes (P450s), members of CYP family, are the most important. P450s are hemethiolate proteins found in all life forms from prokaryotes to eukaryotes. P450s function in the metabolism of aliphatic, alicyclic, and aromatic molecules to regulate hydroxylation, epoxidation, dealkylation, sulfoxydation, deamination, desulphuration, dehalogenation, and noxide reduction [17]. Since CYP65 family includes enzymes can transform terpenoids, members of CYP subfamily in *G*. *clavigera* may play a role in terpene modification. For example, CYP65A hydroxylates an intermediate in the biosynthetic pathway of the sesquiterpenoid mycotoxin trichothecene in *Fusarium species* [18]. CYPome is involved in tolerance of host defensive chemicals as well as stress responses in *G*. *clavigera* [19]. Consistently, our results showed that cytochrome P450 was up-regulated upon berberine treatment, but with no significance, the results of cytochrome P450 from DGE suggested that it might be involved in tolerance of toxic chemicals (Fig 3A and 3B).

**Fig 5:**
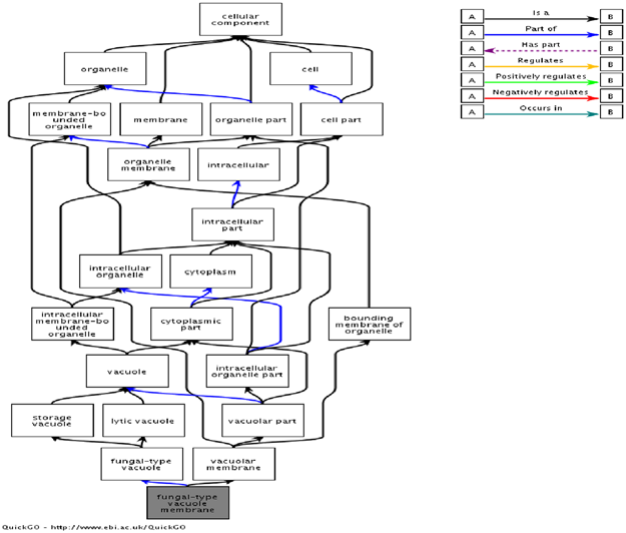
GO term of fungal-type vacuole membrane. Fungal-type vacuole membrane is vacuole membrane, it is the organelle membrane which belongs to cellular component. Being the largest organelles in cells of eukaryotic microorganisms, vacuoles are known to function in intracellular ion homeostasis, storage of metabolites, and degradation of proteins. Vacuoles are important for fungal responses upon berberine chloride treatment. zupT (zinc transporter), the major gene related to fungal type vacuole membrane, is up-regulated upon berberine chloride treatment. Our results suggested that up-regulation of zinc transporter zupT might be related to the compensatory ability of *M*. *canis* upon berberine treatment.

In GO term, fungal-type vacuole membrane is important for the responses of fungi exposed to berberine chloride (Fig 5). Being the largest organelles in cells of eukaryotic microorganisms, vacuoles are known to function in intracellular ion homeostasis, storage of metabolites, intracellular protein transport, and degradation of proteins [20, 21]. Moreover, vacuoles could store organic and inorganic nutrients, which could detoxify the inside toxic substances. In our results, zupT (zinc transporter), the major gene related to fungal type vacuole membrane, is up-regulated upon berberine chloride treatment (Fig 5). Zinc transporters reported a role for zinc in maintaining the cellular balance between cell growth and cell death [21]. Some studies reported that a role for zinc transporter gene expression in zinc-mediated apoptosis and cytoprotection, which are vital following airway inflammation in mouse-lung or cultured-sheep pulmonary artery endothelial cells [22, 23]. In addition, some reports suggested that Zn is required for Akt-dependent cell survival in many types of apoptosis [24, 25]. Furthermore, Zhang’s investigation showed that the induced expressions of ZnT5 and ZnT7 in rat peritoneal mesothelial cells (RPMCs) are essential for the mobilization of zinc into the Golgi under high-glucose conditions, thereby protecting cells from apoptosis [26]. Our results suggested that up-regulation of zinc transporter zupT might be related to the compensatory ability of *M*. *canis* upon berberine treatment.

Post-transcriptional RNA regulons consist of RNP complexes that coordinately regulate the production of functionally related proteins [27, 28]. RNA regulons efficiently and economically coordinate the production of components of biochemical pathways or macromolecular protein complexes in respond to endogenous and exogenous signals. Recent evidence indicates that post-transcriptional partitioning of messenger RNA subsets by RNA-binding proteins help physically localize, temporally coordinate, and efficiently translate cell cycle-related proteins. This dynamic organization of mRNAs encoding cell cycle components contributes to the overall economy of the cell cycle and consistent with the post-transcriptional RNA regulon model of gene expression. From our results, the expression of RNP was down-regulated upon berberine treatment, which means that berberine might inhibit the growth of *M*. *canis* through inhibiting the expression of RNP, the key player in cellular post-transcriptional activity.

## Conclusion

DGE can be used for analyzing variation in transcriptome of the *M*. *canis* cellular responses upon berberine treatment. Our results comprehensively showed the whole gene expression profile of *M*. *canis* upon berberine treatment for the first time. Most of the expression-changed genes were identified to cellular metabolism, biosynthesis, and stress responses. The expressions of glycerol-3-phosphate phosphatase, Na^+^/H^+^ anti-porter, heavy metal tolerance protein, cytochrome P450 enzymes, zinc transporter zupT, and RNP were dramatically changed upon berberine treatment and might play key roles in anti-fungal activity. Our results provided the first step toward a global understanding of the mechanisms that are involved in the exposure of filamentous fungi to azoles. Furthermore, this work demonstrates the potential utility of gene expression profiling in anti-fungal studies.

## Supporting Information

S1 Dataset GO ID and 23 functional groups GO ID and can be categorized into a total of 23 functional groups. (XLSX)

S2 Dataset KEGG database in Microsporum canis DGE class KEGG database in Microsporum canis DGE class, and six terms were significantly enriched (*P* ≤0.05). (XLS)
